# Hematocrit in breast cancer monitoring: an emerging concept in need of evidence

**DOI:** 10.1097/MS9.0000000000003967

**Published:** 2025-10-06

**Authors:** Rameesha Babar, Iqra Ilyas, Muhammad Hussain Mansoor, Tahmed Hassan

**Affiliations:** a Bakhtawar Amin Medical College, Multan, Pakistan; b Nishtar Medical University, Multan, Pakistan; c Enam Medical College, Dhaka, Bangladesh

Breast cancer remains the most diagnosed cancer in women in the United States, accounting for approximately one-third of all new female cancers annually[1]. While advancements in diagnostic and treatment modalities have improved outcomes, long-term monitoring, particularly the early detection of recurrence and metastasis, continues to pose clinical challenges. Conventional surveillance tools, such as imaging, serum-based markers, image-guided biopsy, and lymph-node assessment are often limited by factors such as low sensitivity, high cost, invasiveness, and limited accessibility[2]. This highlights the urgent need for innovative, minimally invasive, and cost-effective tools to improve breast cancer monitoring and provide dynamic, real-time understanding into disease progression and treatment response.

Hematocrit, a routine hematological parameter reflecting the proportion of RBCs in blood, has recently garnered attention as a potential biomarker for tracking disease dynamics in breast cancer[3]. Its ability to reflect systemic physiological changes offers valuable insights into disease monitoring. Hematocrit levels serve as an indirect marker of systemic inflammatory response or bone marrow suppression, particularly in advanced cancer or as a side effect of chemotherapy, thus reflecting the effectiveness of ongoing treatment^[4,5]^. Anemia, often reflected by low hematocrit, is frequently observed in breast cancer patients undergoing chemotherapy or radiation therapy and is associated with reduced quality of life and poorer prognosis. Therefore, early identification of anemia through longitudinal monitoring of hematocrit levels may support timely interventions[6]. Moreover, changes in hematocrit levels offer indirect insights into tumor hypoxia, which can allow for individualized treatment planning[7].

As an easily obtainable, cost-effective, and minimally invasive metric, hematocrit offers a widely accessible tool for longitudinal monitoring, allowing frequent assessments without imposing additional physical or financial burden on patients. Combined analysis of hematocrit levels with established surveillance markers, such as CA 15-3, can provide complementary insights, offering additional layers of information that contribute to a more comprehensive understanding of disease status progression and treatment response[8]. Additionally, integration of hematocrit metrics into AI-driven models may help define clinical patterns and allow precise tracking of the disease.

Hematocrit is also influenced by non-cancer-related factors; therefore, to establish its role in clinical oncology, we must rigorously validate hematocrit’s utility through evidence-based research. Standardizing protocols for measuring hematocrit is essential and should clearly define the clinical context for which hematocrit is being used. Pre-analytical conditions must be controlled, including patients’ hydration status and time of blood collection, to reduce variability[9]. Uniform laboratory techniques or point-of-care (POC) testing devices for real-time monitoring should be adopted[10]. Reference ranges and interpretation thresholds must be stratified according to breast cancer subtypes (e.g., HER2-positive, triple-negative), treatment modalities, disease stages, and patient demographics, as hematocrit levels may vary significantly across these groups[8]. Variability in hematocrit should be analyzed in relation to tumor-specific characteristics and potential confounding factors (e.g., anemia, comorbidities) to determine diagnostic accuracy[9]. Multimodal diagnostic approaches linking hematocrit data with imaging, serum biomarkers, and clinical evaluation will be essential to ensure reliable application. It deems it pivotal to explore the relation between the use of hematocrit, a routine blood investigation, and breast cancer prognosis, further identifying benefits of its use on clinical grounds. Advanced hematocrit innovations reveal its potential to evolve into a dynamic biomarker, providing valuable clinical insights to tackle one of the most critical global healthcare challenges. Broad-scale clinical research should validate its viability in long-term cancer treatment and management. This research was written in accordance with and complies with the TITAN 2025 checklist[11].

## Data Availability

There was no analysis of data done.

## Research registration unique identifying number (UIN)

No human data was collected. Registration to a database was not required.
